# European Ancestry Predominates in Neuromyelitis Optica and Multiple Sclerosis Patients from Brazil

**DOI:** 10.1371/journal.pone.0058925

**Published:** 2013-03-20

**Authors:** Doralina Guimarães Brum, Marcelo Rizzatti Luizon, Antônio Carlos Santos, Marco Aurélio Lana-Peixoto, Cristiane Franklin Rocha, Maria Lucia Brito, Enedina Maria Lobato de Oliveira, Denis Bernardi Bichuetti, Alberto Alan Gabbai, Denise Sisterolli Diniz, Damacio Ramon Kaimen-Maciel, Elizabeth Regina Comini-Frota, Claudia E. Vieira Wiezel, Yara Costa Netto Muniz, Roberta Martins da Silva Costa, Celso Teixeira Mendes-Junior, Eduardo Antônio Donadi, Amilton Antunes Barreira, Aguinaldo Luiz Simões

**Affiliations:** 1 Departamento de Neurociências e Ciências do Comportamento da Faculdade de Medicina de Ribeirão Preto, Universidade de São Paulo, USP, Ribeirão Preto, São Paulo, Brazil; 2 Departamento de Neurologia, Psicologia e Psiquiatria, Faculdade de Medicina de Botucatu, Universidade Estadual Paulista -UNESP, Botucatu, São Paulo, Brazil; 3 Departamento de Genética da Faculdade de Medicina de Ribeirão Preto, Universidade de São Paulo, USP, Ribeirão Preto, São Paulo, Brazil; 4 Departamento de Oftalmologia e Otorrinolaringologia da Faculdade de Medicina, Universidade Federal de Minas Gerais, Belo Horizonte, Minas Gerais, Brazil; 5 Secretaria de Saúde do Estado de Pernambuco, Hospital da Restauração, Recife, Pernambuco, Brazil; 6 Departamento de Neurologia e Neurocirurgia, Universidade Federal de São Paulo, São Paulo, São Paulo, Brazil; 7 Departamento de Clínica Médica, Faculdade de Medicina, Universidade Federal de Goiás, Goiânia, Goiás, Brazil; 8 Departamento de Clínica Médica, Centro de Ciências da Saúde, Universidade Estadual de Londrina, Londrina, Paraná, Brazil; 9 Departamento de Neurologia da Faculdade de Medicina, Universidade Federal de Minas Gerais, Belo Horizonte, Minas Gerais, Brazil; 10 Departamento de Clínica Médica, Divisão de Imunologia Clínica da Faculdade de Medicina de Ribeirão Preto, Universidade de São Paulo-USP, Ribeirão Preto, São Paulo, Brazil; 11 Departamento de Oftalmologia e Otorrinolaringologia e Cirurgia de Cabeça e Pescoço da Faculdade de Medicina de Ribeirão Preto, da Universidade de São Paulo, USP, Ribeirão Preto, São Paulo, Brazil; 12 Departamento de Química, Faculdade de Filosofia, Ciências e Letras de Ribeirão Preto, Universidade de São Paulo, Ribeirão Preto, São Paulo, Brazil; Kyushu University, Japan

## Abstract

**Background:**

Neuromyelitis optica (NMO) is considered relatively more common in non-Whites, whereas multiple sclerosis (MS) presents a high prevalence rate, particularly in Whites from Western countries populations. However, no study has used ancestry informative markers (AIMs) to estimate the genetic ancestry contribution to NMO patients.

**Methods:**

Twelve AIMs were selected based on the large allele frequency differences among European, African, and Amerindian populations, in order to investigate the genetic contribution of each ancestral group in 236 patients with MS and NMO, diagnosed using the McDonald and Wingerchuck criteria, respectively. All 128 MS patients were recruited at the Faculty of Medicine of Ribeirão Preto (MS-RP), Southeastern Brazil, as well as 108 healthy bone marrow donors considered as healthy controls. A total of 108 NMO patients were recruited from five Neurology centers from different Brazilian regions, including Ribeirão Preto (NMO-RP).

**Principal Findings:**

European ancestry contribution was higher in MS-RP than in NMO-RP (78.5% vs. 68.7%) patients. In contrast, African ancestry estimates were higher in NMO-RP than in MS-RP (20.5% vs. 12.5%) patients. Moreover, principal component analyses showed that groups of NMO patients from different Brazilian regions were clustered close to the European ancestral population.

**Conclusions:**

Our findings demonstrate that European genetic contribution predominates in NMO and MS patients from Brazil.

## Introduction

Neuromyelitis optica (NMO) and multiple sclerosis (MS) have been reported in all continents in various distinct populations [1]–[4]. NMO has been referred to as a rare disease which is more frequently observed among non-White individuals. In contrast, MS presents a high prevalence rate, particularly in Whites from Western countries populations, exhibiting a latitudinal gradient variation and being more frequent in Northern areas and less frequent towards Equatorial areas [3], [5]–[7].

Ancestry informative markers (AIMs) have been used as a robust tool to adjust for population admixture, controlling population stratification and avoiding spurious associations in case-control studies [8], [9]. Until now, no study has used AIMs to estimate the genetic contribution of each ancestral population to NMO. In this context, due to its genetically diverse background after five centuries of intense interethnic crossing of individuals of European, African, and Amerindian ancestry, the Brazilian population has been suitable for this proposal. Here we investigated the European, African, and Amerindian genetic ancestry contribution in NMO and MS Brazilian patients.

## Materials and Methods

### Ethics Statement

This study was approved by Ethics Research Committee at Faculty of Medicine of Ribeirão Preto, University of São Paulo, and each subject provided written informed consent.

### Subjects

A total of 128 MS and 108 NMO patients, diagnosed using the McDonald and Wingerchuck criteria, respectively [4], [10], were included in the study. All MS patients were recruited at the University Hospital of the Faculty of Medicine of Ribeirão Preto, University of São Paulo, Brazil, as well as 108 healthy bone marrow donors. NMO patients were recruited from five Neurology centers from different Brazilian regions: 87 from the Southeastern region [58 from Ribeirão Preto (NMO-RP), 12 from the city of São Paulo (NMO-SP), and 17 from the city of Belo Horizonte (NMO-BH)]; seven patients from the Central region (Goiânia, NMO-GO ), and 14 from the Northeastern region (Recife, Pernambuco, NMO-PE). Patients exhibiting Asian ancestry were excluded in the cohort studied. There were no Asian descendants in the MS cohort, and the only four Japanese descendants in the NMO cohort were excluded from the analysis.

### Ancestral Population Genotypes

Genotype data from African (*n* = 128) and European (*n* = 88) populations were kindly provided by Dr. Mark D. Shriver. Brazilian Amerindian genotype data, primarily encompassing representative individuals from *Tikuna* tribe (n = 48), were retrieved from a previous study which described the genotypes of 309 individuals from four Amazon tribes [11].

### Ancestry Informative Markers (AIMs) Selection and Genotyping

Twelve AIMs were selected based on the large allele frequency differences among European, African, and Amerindian populations (Table 1): FY-NULL*1, RB1*1, LPL*1, AT3*1, and APOA*1 discriminate Africans from Amerindians and Europeans; PV92*1, CKM*1, *DRD2-Bcl*I*1, MID-52*1, and MID-575*1 discriminate Amerindians from Africans and Europeans; MID-93*1 differentiates Europeans from Africans and Amerindians; and SB19.3*1 differentiates Africans from Europeans, as previously reported [9]. FY-NULL*1, RB1*1, LPL*1, AT3*1, APOA*1, and PV92*1, and SB19.3*1 were genotyped as previously reported [11]. CKM*1 and *DRD2-Bcl*I*1 single nucleotide polymorphisms were identified using PCR-amplified DNA digested with *TaqI* and *BclI* (New England Biolabs, Ipswitch, MA). MID-52*1, MID-575*1, and MID-93*1 indel polymorphisms were identified using PCR-amplified DNA, followed by direct detection in polyacrylamide gels after silver nitrate staining. Primers were designed using the Primer3 web interface (http://frodo.wi.mit.edu/primer3/).

**Table 1:** Demographic, clinical and laboratory findings of neuromyelitis optica (NMO) and multiple sclerosis (MS) Brazilian patients.

	NMO	MS	p-value
	N = 108	N = 128	
**Gender ratio ( Female/male)**	5.4	2.2	**0.002**
**Age of onset, years (mean** ± SD**)**	35.00 (± 15.25)	30.46 (± 10.52)	**0.01**
**Duration of disease, years (mean ± SD)**	11.05 (± 7.4)	14.82 (± 7.2)	**0.001**
**Laboratory** **NMO-IgG (n = 96)**			
Reagent	61 (63,54%)	–	

### Statistical Analysis

Allele frequency estimates, deviations from Hardy-Weinberg equilibrium expectations and the exact test of population differentiation based on allele or genotype frequencies were performed using GENEPOP software (http://genepop.curtin.edu.au). Significant allele frequency differences were considered when δ values were greater than 0.30. Principal component analysis (PCA) plot was generated based on allele frequencies using MVSP 3.1 software (http://www.kovcomp.co.uk/mvsp/index.html). Ancestry estimates were evaluated based on the gene identity method that takes into account allele frequencies in admixed population in comparison with those observed in ancestral populations, using ADMIX95 program (http://www.genetica.fmed.edu.uy/software.htm).

Multilocus genotypes were used to infer the proportion of the ancestral population contribution to each individual by applying the clustering algorithm implemented at Structure 2.3.3 software (http://pritch.bsd.uchicago.edu/structure.html), and ancestry proportions were represented using triangle plots. The admixture model, correlated allele frequencies, and the following parameters were considered: i) 30.000 burn-in interactions followed by 100.000 additional Markov Chain Monte Carlo interactions, ii) a predefined K  =  3 setting for the number of populations. According to the obtained results, African ancestry indexes (AAI) were estimated for each individual. AAI was expressed as the logarithm of the ratio between the likelihood of a given multilocus genotype occurring in the African population and the likelihood of the multilocus genotype occurrence in the European plus Amerindian populations. Since the variance of AAIs did not follow normal distributions, the non-parametric Kruskal-Wallis test and the Dunn’s multiple comparison post-test were performed using GraphPad Prism software (http://www.graphpad.com/) to compare African ancestry in NMO and MS patients.

## Results

Demographic information for NMO and MS cohorts are presented in **Table 1**. NMO patients were older at disease onset and exhibited shorter disease duration compared with MS patients (p<0.001 and p<0.01, respectively). The female/male ratio was greater in the NMO group (5.4) in comparison with MS (2.2, p<0.0002). Among NMO patients who were screened for NMO-IgG, 63% presented seropositive NMO-IgG (61/96). Twelve patients were not screened.

**Figure 1:**
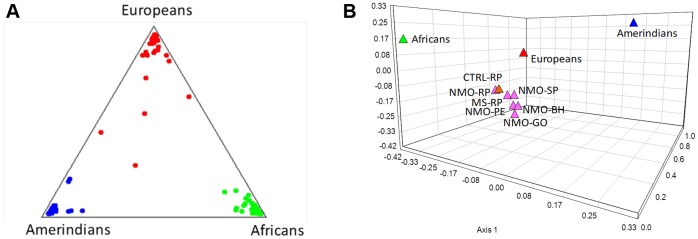
Information of ancestry informative markers (AIMs) set for ancestral populations, multiple sclerosis (MS) and neuromyelitis optica (NMO) patients. (A) The panel of 12 ancestry informative markers (AIMs) for Africans (green), Europeans (red) and Amerindians (blue) were sufficient for an adequate discrimination among ancestral populations. (B) Principal components analysis (PCA) for NMO [Southeastern: Ribeirão Preto (NMO-RP), São Paulo (NMO-SP) and Belo Horizonte (NMO-BH); Central:-Goiânia (NMO-GO), and Northeastern: (Recife-Pernambuco (NMO-PE)] and MS patients from Ribeirão Preto (MS-RP) and control individuals from Ribeirão Preto (CTRL-RP) together with ancestral populations [Africans (green), Europeans (red) and Amerindians (blue)], showing that they clustered closer to Europeans than to Africans and Amerindians.

Allele frequencies for the 12 AIMs in ancestral populations and in NMO, MS, and healthy controls from Ribeirão Preto (CTRL-RP) are shown in **Table 2**. Deviation from the Hardy-Weinberg equilibrium was observed only for the SB19.3 AIM in both MS and NMO patients. The 12 AIMs selected were very informative and able to differentiate ancestral populations. Differences in allele frequencies between ancestral populations exhibited δ values greater than 0.30 (See **Table 2**). The multilocus genotype information of these AIMs was able to discriminate among the ancestral populations. Three totally divergent clusters were obtained without any overlap, each group clustered in one of the vertices of the triangular graph plot (See **Figure 1A**).

**Table 2:** AIMs frequencies observed in MS and NMO patients and healthy controls from Ribeirao Preto (RP), and in Africans (AFR), Europeans (EUR) and Amerindians (AMZ).

			Allele frequency (%)						δ value		
AIMs	Type/Allele^b^	Geneticposition^c^	MS-RP(n = 128)	NMO-RP(n = 58)	Controls-RP(n = 108)	AFR(n = 128)	EUR(n = 88)	AMZ(n = 48)	AFR/EUR	AFR/AMZ	EUR/AMZ
*FY*-NULL*1^a^ (rs2814778)	SNP/G	1q23.2	0.863	0.845	0.850	0.000	0.993	1.000	0.993	1.000	0.007
RB1*1 (rs2252544)	SNP/G	13q14.2	0.355	0.457	0.397	0.920	0.309	0.167	0.611	0.753	0.142
LPL*1 (rs285)	SNP/T	8p21.3	0.569	0.560	0.584	0.980	0.529	0.478	0.451	0.502	0.051
AT3*1 (rs3138521)	Indel/Ins	1q25.1	0.310	0.483	0.421	0.860	0.279	0.021	0.581	0.839	0.258
APOA1*1 (rs3138522)	Alu/Ins	11q23.3	0.883	0.888	0.874	0.453	0.919	0.990	0.466	0.537	0.071
PV92*1 (rs3138523)	Alu/Ins	16q24.1	0.258	0.310	0.336	0.187	0.110	0.935	0.077	0.748	0.825
CKM*1 (rs4884)	SNP/T	19q13.32	0.294	0.371	0.341	0.160	0.287	0.814	0.127	0.654	0.527
*DRD2*-*Bcl*I*1 (rs1079598)	SNP/C	11q23.1	0.165	0.121	0.131	0.087	0.132	0.479	0.045	0.392	0.347
MID-52*1 (rs16344)	Indel/Del	4q24	0.177	0.241	0.182	0.200	0.074	0.755	0.126	0.555	0.681
MID-575*1 (rs140864)	Indel/Ins	1p34.3	0.081	0.078	0.075	0.873	0.993	0.564	0.120	0.309	0.429
MID-93*1 (rs16383)	Indel/Del	22q13.2	0.694	0.716	0.561	0.300	0.816	0.188	0.516	0.112	0.628
SB19.3*1 (rs3138524)	Alu/Ins	19p13.11	0.794	0.698	0.804	0.507	0.904	0.708	0.397	0.201	0.196
			Ancestry estimates								
		European	0.785 ± 0.002	0.687 ± 0.001	0.704 ± 0.012						
		African	0.125 ± 0.001	0.205 ± 0.001	0.179 ± 0.007						
		Amerindian	0.090 ± 0.001	0.108 ± 0.001	0.117 ± 0.009						
			R^2^ = 0.9999	R^2^ = 0.9999	R^2^ = 0.9992						

Significant differences (δ > 0.30) between ancestral populations are underlined in the last columns. European, African and Amerindian ancestry contributions and respective R^2^ values are shown at the bottom of the Table.

a Ancestry informative marker *1 alleles with their reference sequence number from database of National Center for Biotechnological Information (dbSNP/NCBI).

b Single nucleotide polymorphism (SNP), insertion/deletion (Indel), and *Alu* insertion (Alu) polymorphism / allele that characterizes the *1 allele.

c Chromosomal location of each AIM.

The exact test of population differentiation did not reveal differences in allele and genotype frequencies between MS and NMO patients. Similarly, no significant differences were observed when patients were compared to controls. The PCA plot, which unveils similarities and dissimilarities among populations, showed that the cumulative percentage of the variance explained by the first three components was 94.51, which means that 94.5% of the total variance represented by alleles of the 12 AIMs was explained by the present principal component analysis. According to this PCA analysis, all the studied populations clustered together next to the ancestral European population, and were different from African and Amerindian ancestral populations, indicating a closely homogeneous ancestry when evaluated by this set of AIMs (see **Figure 1B**).

Genetic ancestry estimates in patients and controls showed that European contribution was preponderant in all groups, representing 68.7% in NMO and 78.5% in MS patients, whereas African ancestry estimates reached 20.5% for NMO and 12.5% for MS patients (see **Table 2**). These estimates were highly reliable as evaluated by the large R^2^ values. These results are in agreement with the principal component analysis shown in **Figure 1B**, and support the idea that the Brazilian groups studied are highly homogeneous regarding the European ancestry when assessed by this set of 12 AIMs.

**Figure 2:**
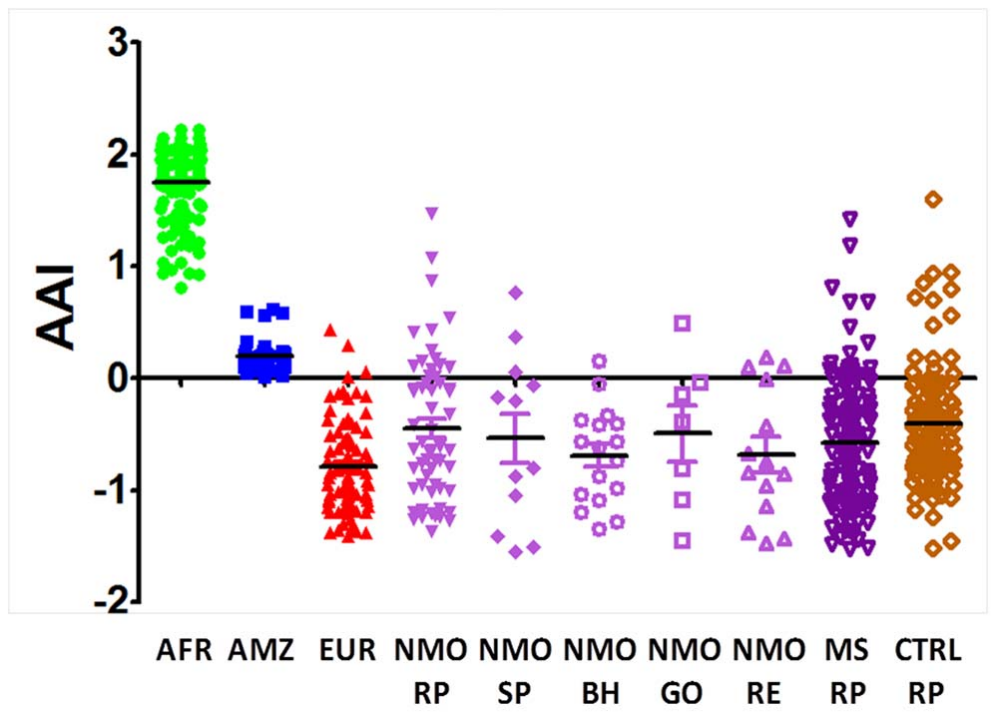
African ancestry indexes (AAI) distribution for ancestral populations, multiple sclerosis (MS) and neuromyelitis optica (NMO) patients. Distribution of African ancestry Index (AAI) for Africans (AFR-green), Amerindians (AMZ-blue) and Europeans (EUR-red) in NMO patients from several Brazilian regions [Southeastern: Ribeirão Preto (NMO-RP), São Paulo (NMO-SP) and Belo Horizonte (NMO-BH); Central:-Goiânia (NMO-GO), and Northeastern: (Recife-Pernambuco (NMO-PE)]; in MS patients from Ribeirão Preto (MS-RP); and in healthy controls from Ribeirão Preto (CTRL-RP).

Considering both ancestral and admixed populations, African ancestry indexes (AAIs) observed for the ancestral African and Amerindian populations differed significantly from all other groups (*p* < 0.05 for each comparison). In addition, AAI values observed for the ancestral European population were different from the MS-RP, NMO-RP, NMO-BH, NMO-PE and CTL-RP (*p* < 0.05 for each comparison), but closely similar to those observed for NMO-GO and NMO-SP (*p* > 0.05 for each comparison). In contrast, AAIs observed for MS-RP, NMO groups and for CTL-RP was closely similar among them (p > 0.05) (see **Figure 2**).

## Discussion

To our knowledge, this is the first study using AIMs to investigate the European, Amerindian, and African genetic ancestry contribution in NMO. The statement that NMO and MS are predominantly associated with either one genetic ancestry or the other is based mainly on our visual perception of phenotype traits from patients and not from the ancestry background. In the present study, we have shown that the contributions of these ancestral groups only present minor differences between NMO and MS patients, and that European contribution predominates in patients of both diseases. Furthermore, the PCA plot showed that NMO groups from different Brazilian regions were clustered close to the European population. In addition, the AAI values for individuals of the NMO groups and of MS-RP did not differ, i.e., their African ancestry was similar. This finding raises questions regarding NMO ancestry, stating that …neuromyelitis optica is relatively common in non-Whites and populations with a minor European contribution to their genetic composition such as Afro-Brazilian [12]. Noteworthy, it is important to emphasize that skin color may not be a reliable marker for genome ancestry, since a previous Brazilian study evaluating 10 AIMs showed that skin color, as determined by physical examination, is a poor predictor of genomic ancestry [13]. In addition, the further evaluation of 40 AIMs in Brazilian subjects from different regions showed that European ancestry was predominant [14].

Despite the small number of AIMs and of small numbers the individuals analyzed, this study discriminated the ancestral groups contributions and indicated that even small number of markers may be sufficient when appropriately selected to answer a specific question. Taken together, these findings support the evidence that phenotypic traits do not reliably reflect the genomic ancestry of NMO and MS individuals

In conclusion, this is the first study demonstrating that the European gene pool predominates in NMO patients. New insights from the contribution of the ancestral populations in NMO and MS patients may support a better understanding of the differential ancestry prevalence in disease and may help advance the use of genomic medicine.
